# Chemical Fingerprint and Multicomponent Quantitative Analysis for the Quality Evaluation of *Cyclocarya paliurus* Leaves by HPLC–Q–TOF–MS

**DOI:** 10.3390/molecules22111927

**Published:** 2017-11-07

**Authors:** Yanni Cao, Shengzuo Fang, Zhiqi Yin, Xiangxiang Fu, Xulan Shang, Wanxia Yang, Huimin Yang

**Affiliations:** 1 College of Forestry, Nanjing Forestry University, Nanjing 210037, China; 182cyn@sina.com (Y.C.); xxfu@njfu.edu.cn (X.F.); shangxulan@njfu.edu.cn (X.S.); yangwanxia@njfu.com.cn (W.Y.); 2 Co-Innovation Center for Sustainable Forestry in Southern China, Nanjing Forestry University, Nanjing 210037, China; 3 Department of Natural Medicinal Chemistry and State Key Laboratory of Natural Medicines, China Pharmaceutical University, Nanjing 10009, China; chyzq2005@126.com (Z.Y.); yanghuimin607@163.com (H.Y.)

**Keywords:** *Cyclocarya paliurus*, fingerprint, HPLC–Q–TOF–MS, quantitative analysis, quality evaluation

## Abstract

*Cyclocarya paliurus* is an edible and medicinal plant containing various bioactive components with significant health benefits. A combinative method using high-performance liquid chromatography (HPLC) fingerprint and quantitative analysis was developed and successfully applied for characterization and quality evaluation of *C. paliurus* leaves collected from 18 geographical locations of China. For the fingerprint analysis, 21 common peaks were observed among the 18 samples, and these peaks were identified by high-performance liquid chromatography coupled with quadrupole time-of-flight mass spectrometry (HPLC–Q–TOF–MS), while a simultaneous quantification of 16 markers was conducted to interpret the variations of contents of these bioactive compounds among the *C. paliurus* leaves from different geographical locations. Quantification results showed that the contents of these sixteen investigated compounds varied greatly among the leaves from different locations. The developed new method would be a valuable reference for further study and development of this bioactive plant.

## 1. Introduction

*Cyclocarya paliurus* is a monotypic genus belonging to the Juglandaceae family, which is mainly distributed in the highlands of subtropical regions in China [1]. *C. paliurus* is commonly called “sweet tea tree” because of the natural sweetness of its leaves. The leaves of *C. paliurus* have long been used as a nutraceutical tea for local people. In addition to being consumed as a nutraceutical tea, it has also been used for drug formulations in traditional Chinese medicine (TCM) or as ingredients of functional foods in China [2,3]. In the past decade, a great deal of modern pharmacological studies have demonstrated that leaves of *C. paliurus* possess biological functions, including enhanced antihypertensive activity, hypoglycemic activity, hypolipidemic activity, improving mental efficiency, anticancer, anti-HIV-1, antibacterial and antioxidant activity [4,5,6,7,8,9,10,11,12,13]. These potential health benefits are attributed to the bioactive compounds in the leaves of *C. paliurus*. The chemical investigations indicated that the leaves of *C. paliurus* contained abundant physiologically active compounds, such as flavonoids, phenolic acids, triterpenoids and polysaccharides [3,6,12,14]. It is noteworthy that several distinctive triterpene compounds named cyclocarioside I, cyclocarioside II, cyclocarioside III, pterocaryoside A, pterocaryoside B and cyclocaric acid B were only isolated and identified from the leaves of *C. paliurus* [14,15,16,17]. Since most botanical extracts have the therapeutic or preventable effects based on the synergic effects of their multiple components and multiple targets, it is insufficient to determine merely several markers of bioactive constituents in the complex botanical extracts [18]. Unfortunately, recent studies on the quality control of *C. paliurus* are only focused on a few selected flavonoids such as isoquercitrin, kaempferol and quercetin or a few selected triterpenoids because many marker components are not commercially available, especially some unique constituents originating from *C. paliurus* [19,20,21]. Obviously, the current analysis on bioactive constituents was insufficient to reflect the complexity and synergistic actions of multiple components in leaves of *C. paliurus.*

Chromatographic fingerprint, a comprehensive and quantifiable identification method, displays the holistic chemical profile of botanical extracts with chromatograms, spectrograms and other graphs by analytical and chemical techniques [22,23,24]. Chromatographic fingerprint can be used to characterize both the marker compounds and the unknown components in a complex sample. It has been widely used in the identification of authenticity, differentiation of origin and evaluation of quality of traditional Chinese medicine and other botanical products [25,26,27,28]. At present, this method has been adopted by the World Health Organization and other authorities as a strategy for quality assessment of botanical products [18,29,30]. Among the chromatographic fingerprinting applied to the authentication and qualitative evaluation of botanical products over the past decade, high-performance liquid chromatography (HPLC) fingerprint emerges to be the most widely used method due to its convenience and efficiency [25,31,32]. To date, little is known about the quality evaluation of *C. paliurus* from different geographic locations. Therefore, measurement of the whole leaf constituents of *C. paliurus* from different geographic regions is of great interest and importance through chromatographic fingerprint analysis and determination of multiple characteristic compounds.

In this present study, 18 leaf samples of *C. paliurus* grown in natural forests were collected in China. Chemical fingerprint of *C. paliurus* was established through a high-performance liquid chromatography coupled with quadrupole time-of-flight mass spectrometry (HPLC–Q–TOF–MS). The chromatograms of the extracted samples from different geographic locations were compared visually and qualitatively analyzed via MS behaviors. Similarity analysis and simultaneous quantification of 16 components in 18 leaf samples of *C. paliurus* were undertaken to evaluate the quality difference among the tested samples from different regions. The aim of this study was to provide comprehensive understandings of chemical profile, quantitative analysis and similarity evaluation of *C. paliurus* leaves from different geographic locations, which could be a valuable reference for further study and development of this plant.

## 2. Results and Discussion

### 2.1. Optimization of Sample Extraction and Chromatographic Conditions

In order to extract the bioactive substances sufficiently and obtain as much fingerprint information as possible, the extraction methods, extraction solvents and extraction time were optimized by using a univariate approach. Compared to refluxing extraction, the ultrasonic method was preferred as it was simpler and more convenient. The samples were extracted using 50%, 70%, 90% or 100% ethanol. By comparing the number of chromatographic peaks and peak areas with the extraction of different solvents, it was clear that, when 70% ethanol was employed, the peak numbers and peak areas reached the highest values. Thus, 70% ethanol was selected as the extraction solvent. The influence of the extraction time under ultrasonication on the efficiency of extraction was also investigated, in which powdered samples were extracted with 70% ethanol for 30, 45 or 60 min. When the ultrasonic extraction time was less than 45 min, the extraction efficiency increased with the time, but the peak areas of the target compounds did not significantly increase after 45 min. The above experiments suggested that samples were optimally extracted by the ultrasonic method with 70% ethanol for 45 min.

In order to obtain the most useful chemical information and better separation in the chromatograms, different HPLC parameters including the column brand, the mobile phase composition, the gradient elution procedure and the detection wavelength were optimized. Three kinds of reversed-phase columns, Phenomenex C18 column (250 mm × 4.6 mm, 5 μm), Waters X-bridge C18 column (250 mm × 4.6 mm, 5 μm) and Waters Sunfire C18 column (250 mm × 4.6 mm, 5 μm) were first evaluated and compared. The Waters X-bridge column was found to be more suitable and provided a better separation of compounds in the leaves, with a more-stable baseline than other brands of C18 columns. Besides, notable differences were observed between methanol–water and acetonitrile–water mobile phase systems. The acetonitrile–water system gave a better resolution than methanol–water. Meanwhile, 0.01% (*v*/*v*) formic acid was added to the mobile phase to improve the resolution and minimize the peak tailing of target compounds. Gradient elution was also used due to the complexity of chemical compositions in the tested leaves. In the process of gradient optimization, gradient time, gradient procedure and initial composition of the mobile phase were taken into consideration. Eventually, a satisfactory separation was attained within 100 min using the optimized gradient elution procedure as described in Section 3.3. However, there is still room to shorten the analytical time to achieve the same fingerprint quality by using other means, such as the faster-speed UPLC. In recent years, UPLC has been demonstrated to be a very powerful tool in chromatographic fingerprinting applications for its high resolution [28]. However, HPLC has emerged as the most widely used method in practice for its easy availability and relatively low price [25,31,32]. With respect to detection wavelength, more detectable common peaks and larger response values could be obtained at 205 nm due to the lack of a chromophore group in the chemical structures of triterpenoids, which show mainly terminal absorptions such as 205 nm in their UV spectra [21,23]. Hence, characteristic chromatographic patterns were obtained by using 205 nm as the detection wavelength.

### 2.2. HPLC Fingerprint Establishment and Similarity Analysis 

**Figure 1:**
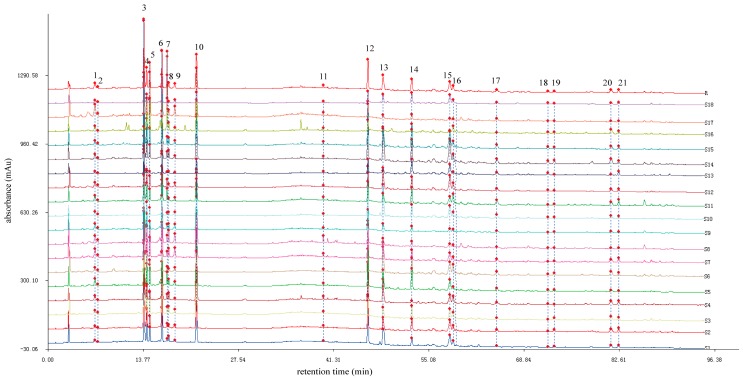
HPLC chromatographic fingerprints of the 18 *C. paliurus* samples and the reference fingerprint (R) obtained by the Similarity Evaluation System for Chromatographic Fingerprint of Traditional Chinese Medicine software (Version 2004A, Chinese Pharmacopoeia Committee, Beijing, China). The peaks marked with 1–21 represent the 21 characteristic common peaks.

The chromatographic fingerprints of 18 leaf samples of *C. paliurus* from different geographic locations are presented in Figure 1. The reference fingerprint (marked with R in Figure 1) was developed with the median of 18 chromatograms to identify and evaluate the quality of *C. paliurus* leaves, and 21 peaks were extracted to be the characteristic common peaks. In general, the characteristic peaks’ selection was based on the criterion that peaks found in each of the chromatograms of samples that originated from different geographic locations were well separated under the given chromatographic conditions, and that they had different relatively large peak areas on different profiles [33].

Similarity analysis was performed to evaluate the resemblance and difference of *C. paliurus* samples. As shown in Figure 1, chromatographic profiles of the tested samples were generally consistent, although the absorption intensity of some peaks and the numbers of peaks were slightly different for some samples. The values of similarity between the generated reference fingerprint and individual sample fingerprints were calculated using the similarity evaluation system. As detailed in Table 1, the similarity values of 12 leaf samples were above 0.9, indicating that similar chemical components were present in these samples regardless of geographic locations. However, relatively low similarity values (less than 0.9) were observed in leaf samples of S6, S10, S13, S14, S16 and S17, suggesting that chemical compositions or contents of the six samples might be different from those with a high similarity value.

### 2.3. Identification of Characteristic Common Peaks in C. paliurus Leaves

**Figure 2:**
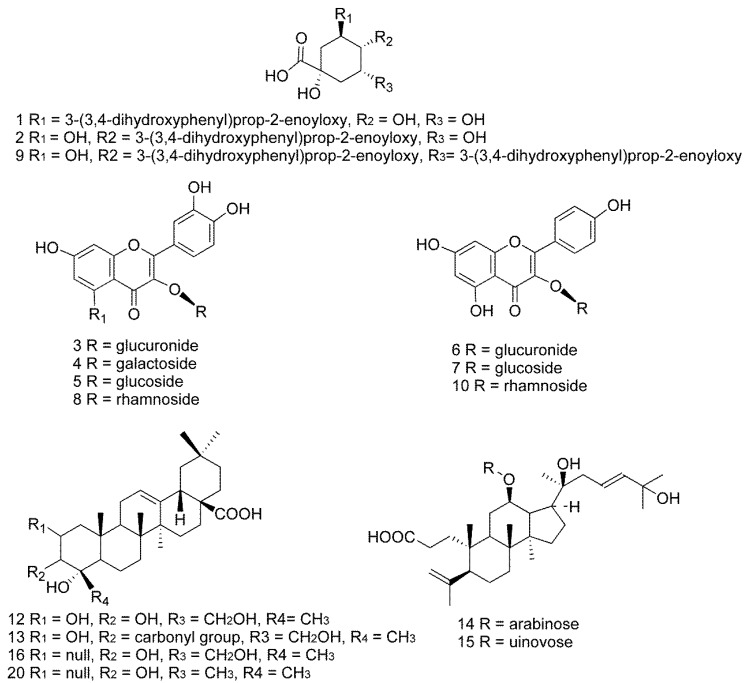
Chemical structures of the 16 quantitative compounds in leaves of *C. paliurus:* (**1**) 3-*O*-caffeoylquinic acid; (**2**) 4-*O*-caffeoylquinic acid; (**3**) quercetin-3-*O*-glucuronide; (**4**) quercetin-3-*O*-galactoside; (**5**) isoquercitrin; (**6**) kaempferol-3-*O*-glucuronide; (**7**) kaempferol 3-*O*-glucoside; (**8**) quercetin-3-*O*-rhamnoside; (**9**) 4,5-di-*O*-caffeoylquinic acid; (**10**) kaempferol-3-*O*-rhamnoside; (**12**) arjunolic acid; (**13**) cyclocaric acid B; (**14**) pterocaryoside B; (**15**) pterocaryoside A; (**16**) hederagenin; (**20**) oleanolic acid.

**Figure 3:**
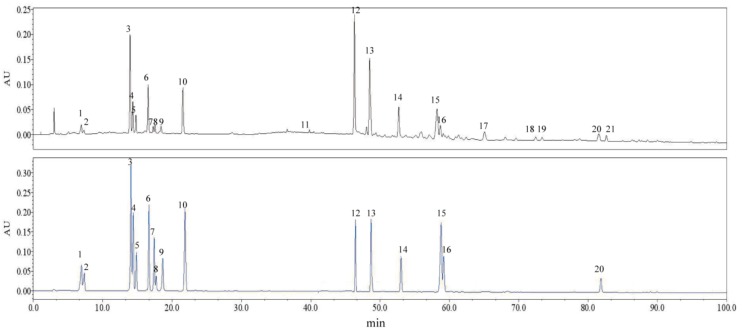
HPLC chromatograms of a representative sample solution (**top**) and a mixed standard solution containing the 16 quantitative compounds (**bottom**).

The 21 characteristic common peaks, which were sufficient to evaluate the quality of *C. paliurus* leaves, were identified using HPLC–Q–TOF–MS. The identification was conducted by comparing HPLC retention time, UV absorption, *m*/*z* of quasi-molecular ions and MS^2^ fragmentation patterns with those of reference substances and some previous reports. In general, quasi-molecular ions of these target compounds were exhibited as [M–H]^−^ in negative ion mode. The data of retention time, MS and MS^2^ fragment ions and the identification results for the peaks labeled in the chromatogram (Figure 1) are summarized in Table 2. Among the 21 characteristic common peaks, three phenolic acids, eight flavonoids and nine triterpenoids were identified or tentatively identified from leaves of *C. paliurus*. Moreover, 16 of these components were unambiguously identified by comparison with their authentic standards’ retention times and MS^2^ data, while others were tentatively inferred based on their chromatographic behaviors and main fragments in MS^n^ spectra, and comparing them with related literature data. The chemical structures of the 16 components are shown in Figure 2, and in addition, the chromatograms of one of the samples and the 16 mixed standards are presented in Figure 3.

#### 2.3.1. Identification of Phenolic Acids

Peaks **1** and **2** were identified as the esters of caffeic acid and quinic acid, respectively, and exhibited a similar [M ‒ H]^−^ ion at *m*/*z* 353 and fragment ions in the MS^2^ scans (Table 2), but eluted at different retention times, which indicated the presence of isomers. Subsequently, they were distinguished as 3-*O*-caffeoylquinic acid and 4-*O*-caffeoylquinic acid compared with their reference standards. While peak **9** showed a [M − H]^−^ ion at *m*/*z* 515, which was 162 Da more than that of peak **2**, fragments at *m*/*z* 191, 179, 173 were the same as compound **2**, suggesting the presence of double esters of caffeic acid. Then, it was further precisely identified as 4,5-di-*O*-caffeoylquinic acid by comparison with its reference compound.

#### 2.3.2. Identification of Flavonoids

Flavonoids in edible and medicinal plants possess a wide range of biochemical and pharmacological effects. Many investigations of *C. paliurus* have demonstrated the presence of some flavonoid compounds in *C. paliurus* leaves [6,12,19]. In the ionization of MS analysis, the flavone aglycone was easily dissociated from the saccharidic residue, resulting in the loss of neutral ion [M − **18**]^−^. Cleavage at the glycosidic *O*-linkages with a concomitant H-rearrangement lead to the elimination of monosaccharide residues, that is, the loss of 162 Da (a hexose unit), 146 Da (a rhamnose unit) and 176 Da (a glucuronosyl group) [34]. A total of eight flavonoid glycosides were identified in the tested samples, which were divided into two types based on their aglycones, namely quercetin and kaempferol derivatives. For example, peak **3** of a [M ‒ H]^−^ ion at *m*/*z* 477 produced a dominant fragment ion at *m*/*z* 301 (Table 2), which was generated by the loss of a glucuronosyl group and assigned to the aglycone ion of quercetin. Thus, peak **3** was identified as quercetin-3-*O*-glucuronide based on its chromatographic behavior and main fragments in MS^n^ spectra compared with literature data [35,36]. Consequently, the peaks of **4**–**8**, **10** and **11** were easily identified by following the principle mentioned above with the details shown in Table 2. Moreover, they were further precisely authenticated by comparison with their reference standards except for the lack of reference standard of peak **11**. By comparing the fragments with the previous literature [36], peak **11** was preliminary supposed to be kaempferol-3-(6′′-(*Z*)-cinnamylglucoside). 

#### 2.3.3. Identification of Triterpenoids

Triterpenoids are another major group of bioactive components from *C. paliurus*. In the present study, six triterpenoids (peaks **12**, **13**, **14**, **15**, **16** and **20**) were unambiguously identified by comparison with their reference standards’ retention time and MS^2^ data, while another three compounds (peaks **17**, **18** and **19**) were tentatively deduced based on their fragmentation pathways and previous reports of *C. paliurus* [14,15,37,38]. Unfortunately, we were unable to identify peak 21.

Since the ingredients in the extracts of the plant are complex, MS confirmation was needed to ascertain that the peaks represented the major compounds of this plant. HPLC–Q–TOF–MS, which provides mass measurement and fragment information, was readily available and reliable for peak identification of constituents in complex extracts of plants. Although negative and positive ion modes were complementary, the negative ion mode was selected, owing to the simplicity and stability of mass spectra, as well as lower background noise.

### 2.4. Method Validation

As shown in Table 3, the method was fully validated and all the 16 analytes demonstrated a good linearity (R^2^ > 0.999) within the test range. The values of LOD and LOQ were all below the level of the test samples except the 4,5-di-*O*-caffeoylquinic acid content of the S13 sample (Table 4), indicating the high detection sensitivity of the method. For the sixteen analytes, the RSD values of the intraday and interday precisions varied from 0.43% to 1.26% and 0.98% to 2.83%, respectively, while the RSD values of repeatability were below 2.13%. The mean recoveries also showed a good range of 96.5–102.9% with RSD less than 3% (Table 3).

### 2.5. Quantification of Chemical Components in C. paliurus Leaves 

The amount of bioactive compounds in *C. paliurus* leaves is very important for the therapeutic effects. Based on the optimized method developed in this study, concentrations of 16 bioactive compounds in leaf samples of *C. paliurus* collected from 18 different geographic locations were determined simultaneously and the results are shown in Table 4, Table 5 and Table 6. The contents of the 16 compounds varied significantly among the 18 samples, whereas the variability was dependent on each individual compound. The differences in the contents of chemical compounds would lead to differences in their efficacies in clinical practice. In terms of phenolic acids, 3-*O*-caffeoylquinic acid showed high concentrations in all samples, especially in the S17 sample collected from Lueyang of Shanxi Province, reaching 2.34 mg/g (Table 4). In addition, the highest sum of the three phenolic acids was also observed in S17, followed by S8 and S7. As for flavonoids, quercetin-3-*O*-glucuronide, kaempferol-3-*O*-glucuronide and kaempferol-3-*O*-rhamnoside were the predominant ingredients in all samples except S16, which was from Qingchuan of Sichuan Province (Table 5). However, S16 had the highest content of kaempferol-3-*O*-glucoside compared with other samples, reaching 2.04 mg/g. Furthermore, S16 was also rich in quercetin-3-*O*-galactoside and isoquercitrin. The total content of the seven flavonoids showed a great variation among different geographical locations. Among the tested samples, the samples from Hefeng of Hubei Province (S8) and Lueyang of Shanxi Province (S17) contained the highest total content (more than 10.0 mg/g), while the sample from Suining of Hunan Province (S13) showed the lowest total amount (less than 2.10 mg/g), indicating that the contents of phenolic compounds in *C. paliurus* leaves may be influenced by environmental factors or genotypes.

Triterpenoids are the typical group of bioactive components from *C. paliurus* leaves. As shown in Table 6, arjunolic acid, cyclocaric acid B, pterocaryoside B and pterocaryoside A were found to be abundant in all samples. In addition, cyclocaric acid B, pterocaryoside B and pterocaryoside A were unique constituents detected from *C. paliurus* leaves, which can be used as characteristic compounds to identify the authenticity of this plant [14,15,16,17]. Our results showed that the samples from Jiangxi Province (S14 and S15) contained the highest levels of the detected triterpenoid components, followed by the samples from Liping of Guizhou province (S6) and Jianghua of Hunan Province (S10). From the view of the content of the six triterpenoid ingredients, samples from Hefeng of Hubei Province (S8), Wufeng of Hubei Province (S9), Qingchuan of Sichuan Province (S16) and Lueyang of Shanxi Province (S17) showed very low triterpenoid contents (Table 6). However, leaf samples from S8 and S17 contained high levels of phenolic acids and flavonoids (Table 4 and Table 5), indicating that the response of various secondary metabolites to environment and genotype might be diverse. 

**Figure 4:**
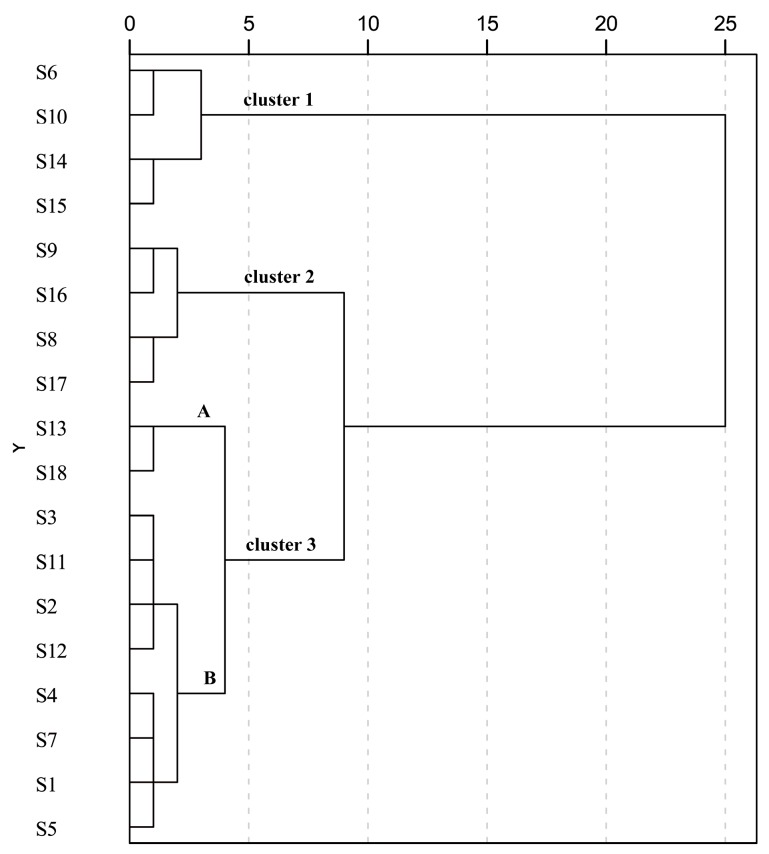
Dendrogram of hierarchical cluster analysis for leaf samples S1–S18 of *C. paliurus* (detailed geographical information for S1–S18 is listed in Table 1). A and B represent two subgroups under cluster 3 based on the squared Euclidean distance.

In order to comprehensively evaluate the quality of *C. paliurus* leaves collected from 18 geographic locations, a hierarchical cluster analysis (HCA) was conducted using the contents of the 16 analytes and the total contents of investigated phenolic acids, flavonoids and triterpenoids as 19 variables. The result indicated that the 18 samples were classified into three distinct groups (Figure 4). Cluster 1 (including S6, S10, S14 and S15) showed high content of the tested triterpenoids. Cluster 2 (including S8, S9, S16 and S17) exhibited relatively high levels of tested phenolic acids or flavonoids, but showed lower levels of detected triterpenoids. However, cluster 3 (including 10 samples) was further divided into two subgroups (A and B, Figure 4). Samples in subgroup A (S13 and S18) showed poor performance in content of both phenolic compounds and triterpenoid compounds, whereas samples in subgroup B (including other samples) showed moderate levels of the investigated compounds. It is worth pointing out that leaf quality in woody medicinal plants could not be evaluated by chromatographic fingerprints alone based on the quantitative data and HCA results from this study. For example, S18 displayed relatively high similarity value (0.936), but the content of the three groups of the major bioactive substances were lower than other samples. On the contrary, S6, S10, S14 and S17 had high content of phenolic acids, flavonoids or triterpenoids regardless of lower similarity values. Hence, a better strategy for comprehensive quality evaluation would be using chromatographic fingerprinting combined with simultaneous quantitative techniques.

Accumulation of phytochemicals could be influenced by numerous internal and external factors, such as environmental conditions, genotypes and silvicultural practices during the growth period [20,39]. The most important environmental factors that affect the quantity and quality of bioactive substance are light, temperature, rainfall, latitude, soil characteristics, altitude and nutrition. Previous studies have demonstrated that light intensity significantly influences flavonoid accumulation in *C. paliurus* [20,40], while genetic variations could be one of the key factors affecting the biosynthesis and accumulation of bioactive compounds and quality of many medicinal plants [19,41,42]. Deng et al. has confirmed that genotype and environment significantly affect the growth and flavonoid accumulation of *C. paliurus*, and the interactions between environment and genotype on the accumulation of flavonoid was also observed [43]. The ancient Chinese “geoherbalism” theory, that the same plant species grown in different geographic regions may differ significantly in the specific bioactive components both qualitatively and quantitatively, also indicates the influence of interaction between environment and genotype on the accumulation of secondary metabolites. Temporal variations in natural compounds are fairly common [23,44]. Fu et al. reported that there are significant seasonal variations in the content of water-soluble polysaccharides of *C. paliurus* leaves [45]. To eliminate the effects of seasonal fluctuation on accumulation of bioactive compounds, the leaves tested in this study were collected at the same time for quantitative and chemical fingerprint analyses. Our results showed that the chemical profiles and contents of the 16 bioactive ingredients varied among the 18 samples from different geographical regions, suggesting that the variations might be attributed to the differences in climatic condition, soil condition and genotype. To reveal the leaf quality difference, additional investigations over the temporal, spatial and genetic variations in *C. paliurus* are needed. 

## 3. Materials and Methods

### 3.1. Plant Materials

18 leaf samples of *C. paliurus* grown in different natural forests were collected and the detailed information is shown in Table 1. At each sampling site, we collected leaves from 6 to 15 dominant trees (based on the size of natural populations) and the leaves were mixed as a batch of sample. All the leaf samples were collected in October of 2014 and were identified for authentication by the corresponding author. A voucher specimen was deposited in the Silviculture Lab of Nanjing Forestry University.

### 3.2. Chemical Reagents and References 

Acetonitrile was of HPLC grade from Tedia (Fairfield, OH, USA); deionized water was further purified by a Milli-Q purification system (Millipore, Millford, MA, USA); HPLC-grade formic acid was purchased from Aladdin Co., Ltd. (Shanghai, China), and other reagents were all of analytical reagent grade. The reference standards of 3-*O*-caffeoylquinic acid, 4-*O*-caffeoylquinic acid, 4,5-di-*O*-caffeoylquinic acid, quercetin-3-*O*-glucuronide, quercetin-3-*O*-galactoside, isoquercitrin, kaempferol-3-*O*-glucuronide, kaempferol-3-*O*-glucoside, quercetin-3-*O*-rhamnoside, hederagenin and oleanolic acid were purchased from Shanghai Yuanye Biotechnology Co., Ltd. (Shanghai, China), and arjunolic acid was purchased from BioBioPha Co., Ltd. (Kunming, China), whereas kaempferol-3-*O*-rhamnoside, cyclocaric acid B, pterocaryoside A and pterocaryoside B were isolated and purified previously from the leaves of *C. paliurus* in the laboratory of China Pharmaceutical University (Nanjing, China) and were elucidated by comparison of spectral data (UV, MS, ^1^H-NMR, ^13^C-NMR) with those of published references [16,17]. The purity of each compound was determined to be more than 98% by normalization of the peak area detected by HPLC–UV.

### 3.3. HPLC Instrument and Chromatographic Conditions

All analyses were carried out on a Waters e2695 Alliance HPLC system (Waters Corp., Milford, MA, USA), equipped with a Waters 2695 separation unit (a quaternary pump solvent management system, an auto sampler, an online degasser, a column heater and a gasket cleaning system), a Waters 2489 ultraviolet detector (UVD), and an Empower 3 data processing system. The chromatographic separation was performed on an X-Bridge C18 column of 250 × 4.6 mm packed with 5 μm particles (Waters Corp., Milford, MA, USA). The mobile phases were composed of (A) water containing 0.01% (*v*/*v*) formic acid and (B) acetonitrile containing 0.01% (*v*/*v*) formic acid with the flow rate of 1.0 mL/min. The column temperature was kept at 45 °C. The wavelength of monitor was set at 205 nm and the injection volume was 10.0 μL. The gradient elution program for *C. paliurus* leaves was carried out as follows: 0–13 min, 8–19% B; 13–28 min, 19–21% B; 28–42 min, 21–50% B; 42–46 min, 50% B; 46–60 min, 50–55% B; 60–64 min, 55–56% B; 64–74 min, 56–66% B; 74–90 min, 66–85% B; 90–95 min, 85–100% B; 95–100 min, 100% B. The post-run equilibration time of gradient elution was 15 min.

### 3.4. HPLC–Q–TOF–MS Confirmation Analysis 

Liquid chromatography (LC)–mass spectrometry (MS) analysis was carried out to confirm the peak identities. The identification was performed on an Agilent 6520 Q–TOF mass spectrometer system equipped with a diode array detector (DAD) and electrospray interface (ESI) (Agilent Technologies, Santa Clara, CA, USA). The MS system was operated in negative ionization modes with the mass scan range set at *m*/*z* 100–1200. The mass spectral parameters were a gas temperature of 300 °C; gas flow of 10 L/min; nebulizer pressure of 30 psi; capillary voltage of 4000 V; cone voltage of 100 V; and collision voltage: 60 V. The chromatographic conditions were same as those described above. Agilent Mass Hunter version B.04.00 software was used for data acquisition and processing. Peaks were identified on the basis of comparison of retention times and MS spectra with standards. 

### 3.5. Sample and Standard Solution Preparation 

All leaf samples of *C. paliurus* were oven-dried at 60 °C until a constant weight was reached. Then, the leaf samples were pulverized into fine powder, and passed through a 60-mesh sieve. The sample extracts were prepared by the method of weight relief [30]. In brief, 0.8 g dried sample powder was accurately weighed and extracted in an ultrasonic cleaning bath (KQ250B, Kunshan Ultrasonic Instruments Co., Ltd., Kunshan, China). Ultrasonication (44 kHz, 500 W) was performed for 45 min with 10 mL 70% (*v*/*v*) ethanol at 70 °C. When the extractions were cooled to room temperature, additional solvent was then added for the complement of weightlessness. After centrifugation at 10,000 rpm for 10 min, all extractions were filtered through a 0.22 μm polytetrafluoroethylene (PTFE) filter prior to HPLC analysis.

Stock solutions were prepared by dissolving accurately weighed standards in absolute methanol. Then, the stock solutions were further diluted to appropriate concentrations for construction of calibration curves. External standard calibrations were established at six data points covering the concentration range of each compound according to the level estimated in the plant samples. The calibration curves were constructed by plotting the peak areas (UV signal) against the concentrations of each analyte. The diluted solutions of the 16 reference compounds were further diluted to a series of concentrations with methanol for obtaining the limits of detection (LOD) and quantification (LOQ). The LOD and LOQ for each marker compound under the present chromatographic conditions were separately determined at signal-to-noise (S/N) ratio of about 3 and 10, respectively. LOD and LOQ for each compound are shown in Table 1. All the solutions were stored in a refrigerator at 4 °C and brought to room temperature before use. All solutions were filtered through 0.22 um filter before injecting into HPLC.

### 3.6. Method Validation 

The system precision was determined by examining six replicate injections of the same sample solution within a day and performing the procedure on three consecutive days. Six independently prepared solutions from the same sample were determined to check the repeatability. The recovery test was conducted to evaluate the accuracy of this method. The powdered sample of *C. paliurus* leaves (0.8 g) was accurately weighed independently six times and spiked with a known amount (approximately equivalent to 1.0 times of the amount of the actual plant leaf sample contained) of the corresponding standard compounds. Then, the spiked samples were extracted and quantified with the methods mentioned above.

### 3.7. Data Analysis 

The evaluation of chromatographic fingerprint was carried out by professional software “Similarity Evaluation System for Chromatographic Fingerprint of Traditional Chinese Medicine” edited by Chinese Pharmacopoeia Committee (Version 2004A, Beijing, China). This software evaluates similarity based on calculations of correlative coefficients for fingerprint chromatographs. Quantitation of sixteen constituents was performed in duplicate for each sample, and the results were expressed as mean ± standard deviation (SD). One-way analysis of variance (ANOVA) was used to compare the contents of the sixteen bioactive compounds in *C. paliurus* among different geographical sources, followed by Duncan’s multiple-range test. The statistical analyses were performed at a 95% confidence level using SPSS 19.0 software (SPSS, Chicago, IL, USA). Additionally, hierarchical cluster analysis (HCA) was conducted to classify samples from different geographic locations by using the contents of 16 analytes and the total contents of phenolic acids, flavonoids and triterpenoids as nineteen variables. In the HCA program, a dendrogram was drawn to characterize the classification results of the samples by Ward’s linkage as the cluster method and squared Euclidean distance as the metric using SPSS 19.0 software.

## 4. Conclusions

In conclusion, a combinative method using HPLC fingerprint and quantitative analysis was first developed and optimized for *C. paliurus* in this study, and the method was validated to be sensitive, accurate and reliable. Based on the established method, obvious variations both in chemical fingerprints and the contents of 16 bioactive ingredients were observed among the 18 samples due to various environmental factors or genotypes. Owing to the tremendous potential of utilizing the leaves of *C. paliurus* for food, medicine and value-added products for human health, the information provided by this study would be of great importance for authenticity identification and quality evaluation of *C. paliurus* leaves in the future.

## Figures and Tables

**Table 1:** The geographical information and similarity values of leaf samples of *C. paliurus.*

Sample No.	Origins	Latitude (N)	Longitude (E)	Similarity
S1	Qimen, Anhui	30°1′11′′	117°31′44′′	0.956
S2	Mingxi, Fujian	26°34′7′′	116°33′46′′	0.987
S3	Pucheng, Fujian	27°55′43′′	118°45′46′′	0.978
S4	Longlin, Guangxi	24°21′36′′	104°34′12′′	0.968
S5	Longsheng, Guangxi	25°22′12′′	109°31′48′′	0.971
S6	Liping, Guizhou	26°20′24′′	109°14′24′′	0.836
S7	Yinjiang, Guizhou	27°44′24′′	108°30′36′′	0.975
S8	Hefeng, Hubei	29°31′12′′	110°15′00′′	0.926
S9	Wufeng, Hubei	30°11′26′′	110°53′52′′	0.957
S10	Jianghua, Hunan	24°55′2′′	112°1′37′′	0.847
S11	Nanzhao, Henan	33°28′35′′	112°00′05′′	0.987
S12	Shangcheng, Henan	31°25′12′′	115°19′12′′	0.917
S13	Suining, Hunan	26°22′24′′	110°7′47′′	0.876
S14	Fenyi, Jiangxi	27°22′12′′	114°18′36′′	0.882
S15	Xiushui, Jiangxi	28°9′7′′	114°31′8′′	0.960
S16	Qingchuan, Sichuan	32°15′00′′	104°30′36′′	0.523
S17	Lueyang, Shanxi	33°22′12′′	105°50′24′′	0.888
S18	Longquan, Zhejiang	27°32′24′′	119°6′36′′	0.936

**Table 2:** Identification of 21 compounds from leaves of *C. paliurus* by developed HPLC–Q–TOF–MS.

Peak No.	t_R_ (min)	[M ‒ H]^−^	MS/MS Fragment Ion (*m*/*z*)	Formula	Identification
**1**	7.4	353.9874	191.0554	C_16_H_8_O_9_	3-*O*-Caffeoylquinic acid
**2**	7.8	353.0875	191.0552;179.0339;135.0444	C_16_H_8_O_9_	4-*O*-Caffeoylquinic acid
**3**	14.9	477.0673	301.0351;178.9979;151.003	C_21_H_18_O_13_	Quercetin-3-*O*-glucuronide
**4**	15.4	463.0888	301.0343;271.0244;178.9979;151.0029	C_21_H_20_O_12_	Quercetin-3-*O*-galactoside
**5**	15.8	463.0888	301.0338;271.0239;178.9981;151.0029	C_21_H_20_O_12_	Isoquercitrin
**6**	17.6	461.9727	286.0431;285.0401;113.0235;85.0296	C_21_H_18_O_12_	Kaempferol-3-*O*-glucuronide
**7**	18.5	447.0935	285.0388;284.0319;255.0295	C_21_H_20_O_11_	Kaempferol-3-*O*-glucoside
**8**	18.8	447.0973	301.0337;285.0388;255.0294;217.0133	C_21_H_20_O_11_	Quercetin-3-*O*-rhamnoside
**9**	19.8	515.1199	353.0864;191.0553;179.0342;135.0443	C_25_H_24_O_12_	4,5-di-*O*-Caffeoylquinic acid
**10**	23.4	431.0981	285.0394;255.0295;227.0342	C_21_H_20_O_10_	Kaempferol-3-*O*-rhamnoside
**11**	40.8	577.136	397.2221;285.0394;163.0389;145.0289	C_30_H_26_O_12_	Kaempferol-3-(6′′-(*Z*)-cinnamylglucoside)
**12**	47.1	487.3429	445.2942;401.3056;389.2698	C_30_H_48_O_5_	Arjunolic acid
**13**	49.7	485.3275	/	C_30_H_46_O_5_	Cyclocaric acid B
**14**	54.2	621.4001	521.3107;489.3571	C_35_H_58_O_9_	Pterocaryoside B
**15**	60.0	635.4162	535.3265;489.3573	C_36_H_60_O_9_	Pterocaryoside A
**16**	60.5	471.3481	145.0286	C_30_H_48_O_4_	Hederagenin
**17**	67.2	621.4017	489.3578;469.3318	C_35_H_58_O_9_	Cyclocarioside J
**18**	74.4	635.4177	489.3579	C_36_H_60_O_9_	Cyclocarioside III
**19**	75.2	603.3894	521.3107;489.3573;471.347	C_35_H_56_O_8_	Cyclocarioside II
**20**	83.5	455.3549	/	C_30_H_48_O_3_	Oleanolic acid
**21**	84.3	277.2192	146.9664; 197.9641;248.9617	C_18_H_30_O_2_	Unknown

**Table 3:** Method validation for simultaneous quantification of 16 constituents in *C. paliurus* leaves.

Compound	Regressive Equation *^a^*	Linear Range (ug/mL)	R^2^	LOD (ng/mL)	LOQ (ng/mL)	Precision		Repeatability RSD% (*n* = 6)	Recovery	
						Intraday RSD% (*n* = 6)	Interday RSD% (*n* = 18)		Mean	RSD% (*n* = 6 )
3-*O*-caffeoylquinic acid	*y* = 4943.9*x* − 37,710	8–640	0.9998	64.25	214.21	0.88	2.14	0.83	98.72	1.89
4-*O*-caffeoylquinic acid	*y* = 6245.2*x* − 14,821	4–192	0.9998	57.93	197.58	0.71	1.94	1.92	96.53	1.24
Quercetin-3-*O*-glucuronide	*y* = 23,136*x* − 119,324	2.5–600	0.9997	40.28	128.74	0.43	1.21	0.53	98.32	2.13
Quercetin-3-*O*-galactoside	*y* = 20,402*x* − 28,339	2–480	0.9994	52.94	174.17	0.48	1.59	0.92	102.43	0.92
Isoquercitrin	*y* = 21,231*x* + 2369.2	2–180	0.9997	58.42	192.52	0.98	2.35	1.89	97.89	2.02
Kaempferol-3-*O*-glucuronide	*y* = 16,702*x* − 92,304	2–640	0.9995	43.98	153.14	0.62	1.93	2.13	100.93	2.61
Kaempferol-3-*O*-glucoside	*y* = 18,066*x* − 87,126	2–480	0.9991	53.85	187.37	1.26	2.78	1.89	99.23	2.67
Quercetin-3-*O*-rhamnoside	*y* = 15,638*x* − 21,262	2–240	0.9993	62.48	199.32	0.49	0.98	0.57	98.92	1.95
4,5-di-*O*-Caffeoylquinic acid	*y* = 9428.7*x* +52,403	2.5–140	0.9997	58.97	201.22	0.63	1.52	0.82	101.29	1.82
Kaempferol-3-*O*-rhamnoside	*y* = 15,648*x* + 12,295	5–150	0.9997	64.13	211.81	0.58	1.68	1.52	97.69	1.91
Arjunolic acid	*y* = 5465.1*x* + 33,575	5–1500	0.9994	78.13	260.17	0.48	1.19	1.04	102.38	2.48
Cyclocaric acid B	*y* = 8579.6*x* − 91,948	7.5–1080	0.9993	90.16	296.36	0.92	2.83	2.05	102.9	2.52
Pterocaryoside B	*y* = 3716.1*x* + 52,647	6.7–1608	0.9991	72.46	210.27	1.23	2.42	2.04	98.19	2.61
Pterocaryoside A	*y* = 3746.1*x* + 56,466	10–1200	0.9991	86.39	268.62	0.84	1.96	1.29	102.85	1.85
Hederagenin	*y* = 6054.7*x* + 25,521	8–640	0.9997	72.82	232.3	0.91	2.53	1.83	101.83	1.69
Oleanolic acid	*y* = 7144.8*x* + 963.97	1–200	0.9994	58.41	185.72	1.23	2.59	2.43	98.28	2.93

*^a^* *y* is the peak area, while *x* is the concentration of each analyte (ug/mL).

**Table 4:** Quantitative determination of three phenolic acids in 18 samples of *C. paliurus* leaves *^a^*.

Sample No.	Content (mg g^−1^)			
	3-*O*-Caffeoylquinic Acid	4-*O*-Caffeoylquinic Acid	4,5-di-*O*-Caffeoylquinic Acid	TPC *^b^*
S1	0.50 ± 0.03j	0.15 ± 0.00i	0.11 ± 0.00l	0.77 ± 0.04h
S2	0.52 ± 0.00j	0.21 ± 0.01h	0.17 ± 0.00hi	0.91 ± 0.00g
S3	0.80 ± 0.04hi	0.32 ± 0.01fg	0.22 ± 0.01f	1.34 ± 0.07f
S4	0.82 ± 0.00ghi	0.11 ± 0.01j	0.08 ± 0.00m	1.01 ± 0.01g
S5	1.31 ± 0.00d	0.29 ± 0.01g	0.30 ± 0.01d	1.90 ± 0.02c
S6	0.89 ± 0.02fg	0.37 ± 0.01e	0.25 ± 0.00e	1.52 ± 0.02e
S7	1.54 ± 0.01b	0.50 ± 0.02c	0.62 ± 0.00b	2.66 ± 0.03b
S8	1.42 ± 0.01c	0.67 ± 0.00a	0.63 ± 0.03b	2.71 ± 0.02b
S9	1.00 ± 0.01e	0.34 ± 0.02f	0.32 ± 0.02c	1.66 ± 0.04d
S10	0.78 ± 0.04i	0.31 ± 0.02g	0.18 ± 0.00hi	1.27 ± 0.11f
S11	1.00 ± 0.05e	0.22 ± 0.01h	0.14 ± 0.01jk	1.37 ± 0.07f
S12	0.94 ± 0.00ef	0.44 ± 0.02d	0.21 ± 0.00fg	1.60 ± 0.01de
S13	0.46 ± 0.00j	0.12 ± 0.00j	Trace *^c^*	0.58 ± 0.01i
S14	0.92 ± 0.01ef	0.22 ± 0.00h	0.16 ± 0.01ij	1.31 ± 0.06f
S15	0.81 ± 0.04ghi	0.30 ± 0.01g	0.19 ± 0.01gh	1.31 ± 0.09f
S16	0.89 ± 0.03fgh	0.13 ± 0.00ij	0.22 ± 0.00f	1.25 ± 0.03f
S17	2.34 ± 0.00a	0.62 ± 0.03b	0.66 ± 0.01a	3.61 ± 0.01a
S18	0.54 ± 0.03j	0.22 ± 0.01h	0.12 ± 0.01kl	0.88 ± 0.08gh

*^a^* Data are expressed as mean ± SD of duplicate experiments. Different letters indicate significant differences among the *C. paliurus* from different geographic regions in phenolic acid content (*p* ≤ 0.05 by Duncan’s test); *^b^* TPC: contents of total three phenolic acids; *^c^* Trace: under quantification limit (LOQ).

**Table 5:** Quantitative determination of seven flavonoids in 18 samples of *C. paliurus* leaves *^a^*.

Sample No.	Content (mg g^−1^)							
	Quercetin-3-*O*-glucuronide	Quercetin-3-*O*-galactoside	Isoquercitrin	Kaempferol-3-*O*-glucuronide	Kaempferol-3-*O*-glucoside	Quercetin-3-*O*-rhamnoside	Kaempferol-3-*O*-rhamnoside	TFC *^b^*
S1	2.15 ± 0.06c	0.79 ± 0.02ab	0.37 ± 0.02f	1.16 ± 0.00gh	0.19 ± 0.00jk	0.21 ± 0.00e	1.48 ± 0.02f	6.35 ± 0.12de
S2	1.60 ± 0.05d	0.52 ± 0.02d	0.31 ± 0.01g	1.13 ± 0.01h	0.22 ± 0.01i	0.14 ± 0.00gh	1.04 ± 0.02h	4.95 ± 0.12g
S3	2.09 ± 0.11c	0.71 ± 0.04c	0.55 ± 0.02c	1.19 ± 0.06gh	0.27 ± 0.01fg	0.24 ± 0.01d	1.26 ± 0.06g	6.29 ± 0.38de
S4	2.14 ± 0.01c	0.42 ± 0.01f	0.20 ± 0.01i	1.39 ± 0.03e	0.24 ± 0.00h	0.24 ± 0.00d	2.00 ± 0.14c	6.63 ± 0.15de
S5	1.67 ± 0.01d	0.55 ± 0.00d	0.51 ± 0.00d	1.15 ± 0.00h	0.43 ± 0.00c	0.22 ±0.00e	2.17 ± 0.08b	6.70 ± 0.06d
S6	0.79 ± 0.01g	0.53 ± 0.00d	0.30 ± 0.00gh	0.81 ± 0.01j	0.20 ± 0.01ijk	0.11 ± 0.01i	0.81 ± 0.00i	3.55 ± 0.06h
S7	2.56 ± 0.01b	0.82 ± 0.01a	0.51 ± 0.01d	1.30 ± 0.01ef	0.25 ± 0.01gh	0.31 ± 0.00b	1.73 ± 0.01d	7.48 ± 0.02c
S8	3.98 ± 0.09a	0.46 ± 0.00e	0.53 ± 0.01cd	2.30 ± 0.08a	0.30 ± 0.02de	0.43 ± 0.02a	2.63 ± 0.1a	10.63 ± 0.33a
S9	2.11 ± 0.09c	0.40 ± 0.01fg	0.19 ± 0.01i	1.64 ± 0.01d	0.21 ± 0.01ij	0.19 ± 0.01f	1.57 ± 0.05ef	6.32 ± 0.10de
S10	1.09 ± 0.05f	0.38 ± 0.02g	0.21 ± 0.01i	0.83 ± 0.04ij	0.16 ± 0.01l	0.15 ± 0.01gh	0.82 ± 0.041i	3.65 ± 0.18h
S11	1.22 ± 0.06e	0.44 ± 0.02ef	0.29 ± 0.01gh	1.17 ± 0.06gh	0.42 ± 0.02c	0.12 ± 0.01hi	1.16 ± 0.16gh	4.81 ± 0.24g
S12	0.86 ± 0.01g	0.41 ± 0.01fg	0.28 ± 0.01h	1.25 ± 0.01fg	0.30 ± 0.00de	0.13 ± 0.00h	0.57 ± 0.01j	3.80 ± 0.02h
S13	0.50 ± 0.00h	0.11 ± 0.00h	0.05 ± 0.00k	0.67 ± 0.01k	0.11 ± 0.01m	0.08 ± 0.00j	0.55 ± 0.01j	2.07 ± 0.02i
S14	1.07 ± 0.03f	0.78 ± 0.01b	0.37 ± 0.01f	1.68 ± 0.06d	0.28 ± 0.01ef	0.30 ± 0.00b	1.71 ± 0.01de	6.20 ± 0.06e
S15	1.64 ± 0.07d	0.69 ± 0.03c	0.44 ± 0.02e	1.95 ± 0.10c	0.31 ± 0.02d	0.25 ± 0.01cd	1.26 ± 0.10gh	6.56 ± 0.48de
S16	0.16 ± 0.00i	0.80 ± 0.00ab	0.74 ± 0.00b	0.57 ± 0.00l	2.04 ± 0.00a	0.26 ± 0.00c	1.23 ± 0.00g	5.80 ± 0.00f
S17	2.15 ± 0.01c	0.56 ± 0.01d	1.27 ± 0.01a	2.21 ± 0.01b	1.81 ± 0.01b	0.22 ± 0.00e	1.94 ± 0.00c	10.15 ± 0.05b
S18	0.58 ± 0.03h	0.13 ± 0.01h	0.10 ± 0.01j	0.91 ± 0.05i	0.18 ± 0.01kl	0.06 ± 0.00j	0.38 ± 0.05k	2.36 ± 0.11i

*^a^* Data are expressed as mean ± SD of duplicate experiments. Different letters indicate significant differences among the *C. paliurus* from different geographic regions in flavonoid content (*p* ≤ 0.05 by Duncan’s test); *^b^* TFC: content of total seven flavonoids.

**Table 6:** Quantitative determination of six triterpenoids in 18 samples of *C. paliurus* leaves *^a^*.

Sample No.	Content (mg g^−1^)						
	Arjunolic Acid	Cyclocaric Acid B	Pterocaryoside B	Pterocaryoside A	Hederagenin	Oleanolic Acid	TTC *^b^*
S1	5.52 ± 0.23c	1.00 ± 0.05f	1.61 ± 0.081i	3.78 ± 0.09d	1.02 ± 0.00hi	0.26 ± 0.01h	13.18 ± 0.49e
S2	3.13 ± 0.16f	1.06 ± 0.02f	2.62 ± 0.13g	3.82 ± 0.06d	0.96 ± 0.01i	0.40 ± 0.00e	11.99 ± 0.53fg
S3	2.22 ± 0.11g	0.89 ± 0.04g	2.62 ± 0.13g	3.18 ± 0.16f	1.14 ± 0.06fg	0.45 ± 0.02d	10.50 ± 0.20h
S4	4.29 ± 0.15d	1.82 ± 0.02c	0.46 ± 0.03k	2.89 ± 0.14fg	1.32 ± 0.03cd	0.46 ± 0.00d	11.24 ± 0.20gh
S5	4.29 ± 0.15d	1.51 ± 0.01e	4.70 ± 0.12c	3.49 ± 0.09e	0.65 ± 0.03k	0.17 ± 0.00i	14.79 ± 0.45d
S6	5.77 ± 0.29bc	2.17 ± 0.00b	3.46 ± 0.00d	4.88 ± 0.04c	1.75 ± 0.01a	0.54 ± 0.00c	18.57 ± 0.54c
S7	3.92 ± 0.04e	1.45 ± 0.02e	2.20 ± 0.11h	2.95 ± 0.07f	0.74 ± 0.04j	0.37 ± 0.00f	11.62 ± 0.34fgh
S8	2.11 ± 0.11g	0.70 ± 0.02h	1.10 ± 0.02j	1.39 ± 0.06i	0.45 ± 0.01m	0.10 ± 0.00jk	5.85 ± 0.21k
S9	2.13 ± 0.11g	0.68 ± 0.00hi	0.95 ± 0.00j	1.05 ± 0.05j	0.41 ± 0.02m	0.16 ± 0.00i	5.38 ± 0.41kl
S10	5.98 ± 0.02b	2.86 ± 0.14a	2.99 ± 0.15f	4.91 ± 0.25c	1.10 ± 0.06gh	0.41 ± 0.02e	18.25 ± 1.17c
S11	2.02 ± 0.10g	0.85 ± 0.04g	3.10 ± 0.16ef	2.65 ± 0.13g	1.70 ± 0.09a	0.40 ± 0.02e	10.72 ± 0.57h
S12	1.66 ± 0.08h	1.63 ± 0.00d	2.89 ± 0.11f	4.81 ± 0.04c	1.40 ± 0.03c	0.29 ± 0.00g	12.67 ± 0.26ef
S13	1.62 ± 0.01h	0.59 ± 0.01i	3.31 ± 0.13de	3.17 ± 0.14f	0.40 ± 0.00m	0.11 ± 0.00j	9.19 ± 0.26i
S14	6.69 ± 0.26a	2.80 ± 0.06a	5.16 ± 0.14b	8.38 ± 0.16a	1.52 ± 0.08b	0.67 ± 0.00a	25.22 ± 0.30a
S15	5.47 ± 0.27c	1.90 ± 0.10c	5.61 ± 0.28a	6.06 ± 0.30b	1.26 ± 0.02de	0.36 ± 0.01f	20.67 ± 1.03b
S16	1.39 ± 0.07h	0.39 ± 0.00j	0.64 ± 0.03k	0.67 ± 0.03k	1.20 ± 0.05ef	0.59 ± 0.02b	4.89 ± 0.15kl
S17	1.67 ± 0.08h	0.43 ± 0.01j	1.17 ± 0.03j	0.73 ± 0.00k	0.28 ± 0.00n	0.08 ± 0.00k	4.36 ± 0.22l
S18	1.35 ± 0.07h	0.68 ± 0.03hi	2.06 ± 0.10h	2.27 ± 0.11h	0.54 ± 0.03l	0.17 ± 0.00i	7.08 ± 0.35j

*^a^* Data are expressed as mean ± SD of duplicate experiments. Different letters indicate significant differences among the *C. paliurus* from different geographic regions in triterpenoid content (*p* ≤ 0.05 by Duncan’s test); *^b^* TTC: content of total six triterpenoids.
